# Z-Bar Shoeing Demonstrates Potential for Long-Term Foot Pain Management during an Exercise Training Regimen in a Show Jumping Pony with Uniaxial Palmar Pain

**DOI:** 10.1155/2022/8468403

**Published:** 2022-04-13

**Authors:** Kanokpan Sanigavatee, Chanoknun Poochipakorn, Ponlakrit Charoenchanikran, Weena Joongpan, Metha Chanda

**Affiliations:** ^1^Veterinary Clinical Studies Program, The Graduate School, Kasetsart University Kamphaeng Saen Campus, Nakhon Pathom, Thailand; ^2^29th Cavalry Squadron Royal Horse Guard, King's Guard, 2nd Cavalry Brigade, Royal Thai Army, Bangkok, Thailand; ^3^Faculty of Veterinary Medicine, Rajamangala University of Technology Tawan-Ok, Chonburi, Thailand; ^4^Department of Large Animal and Wildlife Clinical Science, Faculty of Veterinary Medicine, Kasetsart University Kamphaeng Saen Campus, Nakhon Pathom, Thailand

## Abstract

Z-bar shoeing has been implemented to relieve uniaxial palmar pain arising from the structures in the affected region. However, there have been no reports on the long-term application of the z-bar shoe during exercise training regimens. A 10-year-old mixed-breed show jumping pony presented with an occasional short stride and abnormal rhythm while turning during routine exercise for three months. Gait analysis conducted by trotting off on both hard and soft surfaces showed no lameness in the straight line on both types of surfaces. However, right forelimb lameness was detected with moderate and slight pain accompanying hard surface lunging in clockwise and counterclockwise directions, respectively. Sequential examination of uniaxial perineural anaesthesia confirmed that the pony suffered from medial palmar pain on the right foreleg. Mild distal border irregularity of the navicular bone was also observed radiographically. The z-bar shoe was designed relative to the palmar digital anaesthesia and subsequently applied on the lame leg. The pony demonstrated a marked reduction in lameness severity immediately post-Z-bar shoeing. Physical exercise was resumed a few days after the shoeing practice. The pony underwent routine exercise training while continuously fitting with the Z-bar shoe for 24 weeks without recurrent lameness or complications. Application of z-bar shoe showed the potential for long-term foot pain management during an exercise training regimen in a show jumping pony with uniaxial palmar pain.

## 1. Introduction

Palmar hoof pain has been described as a common cause of lameness, accounting for one-third of forelimb lameness in horses [1, 2]. The palmar pain is derived from several pathological lesions of the bone and associated soft tissues localised in the hoof's caudal region [3, 4]. Common disorders contributing to palmar hoof pain include those involving the navicular bone and its apparatus, navicular bursitis, distal deep digital flexor tendinitis, laminar tearing or bruising on the palmar region, distal phalanx damage, and hoof distortion [2]. Lameness examination had been traditionally performed to investigate evidence of foot pain [5, 6]. Apart from gait analysis, palmar digital nerve anaesthesia is effectively implemented to localise the palmar pain [7, 8]. Diagnostic imaging is subsequently applied complementary to the regional anaesthesia for accurate diagnosis and appropriate individualised treatment [3, 9]. However, determining the source of pain arising from structures in the palmar hoof capsule is still challenging [3, 10]. In addition, lesions visualised using diagnostic tools may be observed irrespective of the lameness [2, 11]. Hence, the practitioner must carefully evaluate and manage the existing foot pain.

In conjunction with various corrective trimming and shoeing, the medical intervention has been designed to eliminate the palmar pain related to the structural defects causing the pain [2, 4, 12, 13]. The z-bar shoe provides the advantage of managing palmar hoof crack [14] or subsolar abscess at the palmar region [15]. Recently, z-bar shoeing with sequentially uniaxial perineural anaesthesia has been successfully implemented for palmar hoof pain management without medical administration [16]. It also assists in correcting the shear heels' condition in the affected horse's hooves [17]. Despite providing a positive outcome for hoof pain management, long-term application of the z-bar shoe during an exercise training regimen for horses with foot pain has not been studied. This report describes the potential of long-term Z-bar shoe application to manage foot pain during an exercise training regimen in a show jumping pony with uniaxial palmar pain.

## 2. Case Description

A 10-year-old mixed-breed show jumping pony (mare, weight 412 kg, height 143 cm) presented with intermittent stride abnormalities while turning sharp curves during exercise. In the owner's possession for four years, the pony had demonstrated these abnormalities for three months. The pony had been participating in an exercise training regimen for show jumping, including 50 min aerobic exercise, five or six days a week, and a jumping session two or three days a week. For approximately three months, it had been participating in show jumping classes that included 90–120 cm high obstacles. The pony was housed in a 4 × 3 m^2^ individual stable and fed a commercial pellet four times daily. Unlimited clean water was also available in its stable. No medication or another intervention was administered before presenting to the practitioner.

**Figure 1:**
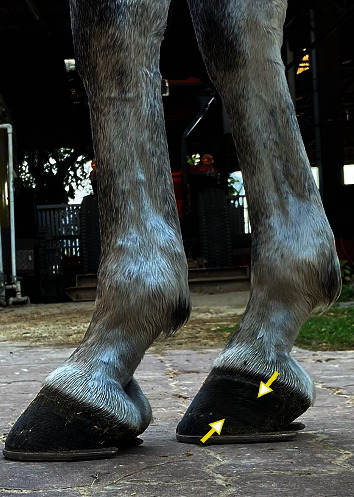
The lateral view image demonstrates a normal hoof-pastern axis in the right foreleg but broken back conformation in the left foreleg. The growth rings were also exhibited in the right hoof wall's medial side (white head of yellow arrows).

Upon arrival, the pony exhibited normal behaviour, and the physical examination yielded no abnormal findings. Gait analysis included trotting and lunging on both soft and hard surfaces. Lameness was graded according to the scale developed by the American Association of Equine Practitioners (AAEP) [18], in which 0 indicated no lameness and 5 represents non-weight-bearing. The pony demonstrated no foot pain following trotting off in a straight line on both hard and soft surfaces (Supplemental Files 1 and 2). No lameness was detected after lunging on the soft surface in clockwise and counterclockwise directions. It demonstrated obvious lameness on the right foreleg with moderate (3/5) and slight (1/5) pain accompanying clockwise and counterclockwise lunging, respectively (Supplemental Files 3 and 4). The pony showed no sensitivity to the hoof tester following an intermittent compression on the entire right front hoof's solar surface. Palpation, joint flexion, and extension tests performed consecutively on the lame leg showed no abnormal findings. In addition, no conformation defects such as long-toe low-heel, sheared heel, and horn tubule degradation were visualised on the right foreleg's hoof capsule. However, growth rings appeared in the hoof wall's medial aspect (Figure 1).

**Figure 2:**
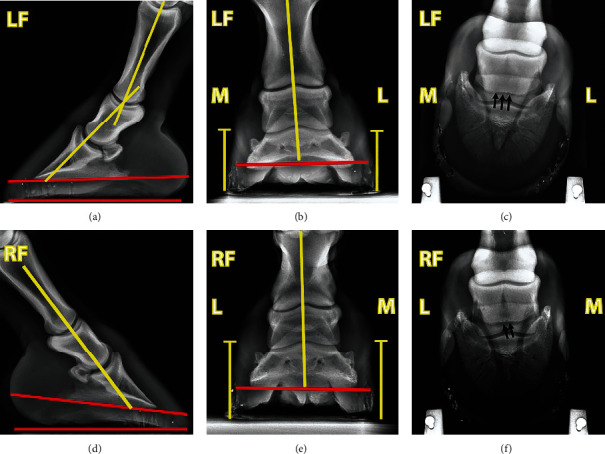
Radiographic images of the (a–c) left and (d–f) right front hooves of a show jumping pony. Both front hooves demonstrated a positive hoof angle, denoted by the angle between red lines at the ventral aspect of the coffin bone and ground contact surface (a, d), with a greater degree in the right front hoof (d). The left front hoof also showed a negative hoof-pastern axis (a). The hoof wall height between the medial and lateral sides of each hoof was equal, indicating no shear heels were observed in both front hooves (b, e). A slight irregularity of the navicular bone's ventral border was concomitantly detected in both front hooves (c, f).

Sequentially uniaxial perineural anaesthesia using 2% lidocaine hydrochloride (L.B.S. Laboratory Ltd. Bangkok, Thailand) was administered to identify pain location according to a method described previously [16, 17]. Briefly, the pony underwent uniaxial local anaesthesia on the right forelimb. The injection was originated from lateral palmar digital nerve desensitisation. The pony was further lunged in both directions on the hard surface to evaluate the lameness score in response to each point of the desensitisation. The local anaesthesia was terminated if no lameness was detected. There was no change in lameness score in response to the lateral palmar digital nerve anaesthesia on the right front leg (Supplemental File 5). The clockwise lunging lameness score reduced significantly, from 3 to 1 out of 5, after medial palmar digital anaesthesia (Supplemental File 6), and there was almost no lameness while lunging in the counterclockwise direction (Supplemental File 7). Further desensitisation controlled at the medial abaxial nerve resulted in no lameness on the right foreleg in both lunging directions (Supplemental Files 8 and 9). Radiographic projections of lateromedial, dorsopalmar, and 65° dorsopalmar positions showed minor irregularity of the navicular bone's distal border in both front hooves (Figure 2). Since there was little evidence specifying pain localisation, the pony was provisionally diagnosed with medial palmar pain in the right forelimb. The differential diagnosis included medial collateral ligament desmitis of the distal interphalangeal (DIP) joint, lamina tearing and associated medial hoof wall disease, and other soft tissue injuries in the affected palmar region. The treatment procedure for the collateral ligament desmitis or other soft tissue injuries at the palmar region consists of stall rest, with controlled exercise, analgesic and anti-inflammatory medication, and shoeing [2, 19].

**Figure 3:**
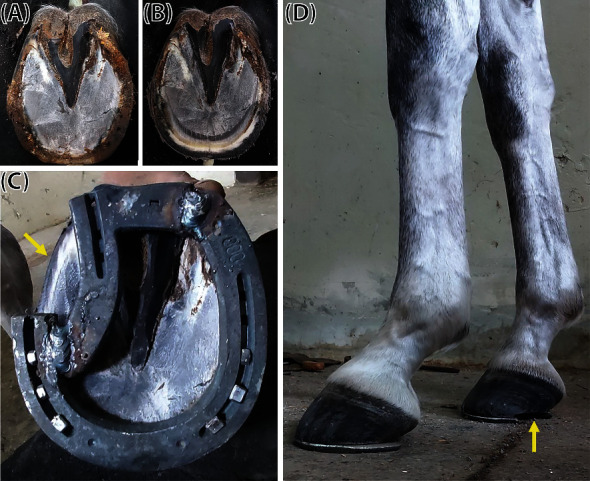
The application of the z-bar shoe on the affected right front hoof. The hoof was trimmed and rasped before fitting with the z-bar shoe (a, b). A non-weight-bearing portion was created on the medial side of the shoe base on the local anaesthesia area (yellow arrow) (c, d). Hoof conformation was adjusted bilaterally to increase the hoof angle and improve the hoof-pastern axis position (d).

**Figure 4:**
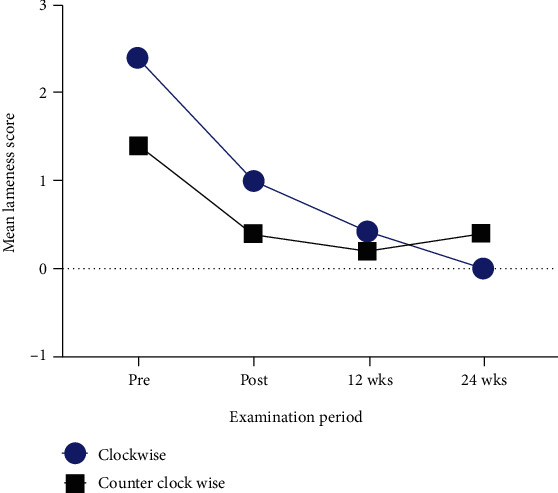
The mean lameness score on the right forelimb following the hard surface lunging in the clockwise and counterclockwise directions. Gait analysis was performed before shoeing, immediately postshoeing, and 12 weeks and 24 weeks postshoeing.

**Figure 5:**
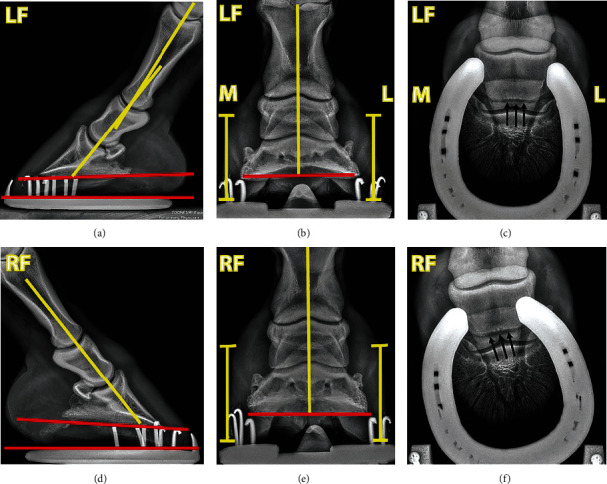
Radiographic images of the (a–c) left and (d–f) right front hooves of a show jumping pony at 30 weeks of shoeing management. The left front hoof demonstrated an improvement in the hoof-pastern axis after a given period of management (a), while a positive hoof angle was still observed in the right front hoof but displayed a lower degree than before shoeing management (d). Hoof's balance was also corrected in both front hooves (b, e). A slight irregularity of the navicular bone's ventral border was still marked in both front hooves (c, f).

A z-bar shoe was employed to manage the pony's chronic foot pain. It was created from the conventional metal shoe (Mustadfors Bruks, Dals Långed, Sweden) according to the pain location, similar to a previously described method [16, 17]. A non-weight-bearing portion of the z-bar shoe was designed to eliminate loading on the medial palmar region (Figures 3(a)–3(c)). The pony was scheduled for the z-bar shoeing at four-week intervals for 24 weeks. The severity of lameness was further evaluated by five experienced clinicians before shoeing, immediately after shoeing, and 12 and 24 weeks postshoeing. Notably, the pony showed a marked lameness score improvement immediately after shoeing (Supplemental Files 10 and 11). The lameness score improvement remained until the end of observation at 24 weeks postshoeing (Supplemental Files 12 and 13) (Figure 4). The radiographic images, taken at 30 weeks of the management period, also showed an improvement of hoof balance in both front hooves (Figure 5).

The pony was assigned to perform light to moderate exercise regimen a few days after the first z-bar shoeing. The initial aerobic exercise program consisted of 5 min walking, 10 min trotting, and 5 min light cantering 3–4 days a week. Comprehensive aerobic exercise training, including 10 min walking, 20 min trotting, and 10 min cantering, was resumed two weeks after initial shoeing. The exercise capability during the exercise session was determined by equipping the pony with a heart rate detector device (Seaver, Paris, France) for monitoring the heart rate, training intensity, speed, and distance and analysing gait (Supplemental File 14). The parameters recorded during the exercise training at 12, 16, 20, and 24 weeks of shoeing are shown in Table 1. The pony exhibited competent trotting and cantering during the exercise sessions (Supplemental File 15). It was exercised at low training intensity and appropriate duration for the aerobic training regimen. Moreover, normal cadence and elevation ranges were maintained during the exercise training. Despite a slight asymmetry during trotting at 12–20 weeks of shoeing, the trotting symmetry was restored following strategic adjustment of the hoof conformation at 24 weeks of shoeing (Figure 3(d)) (Supplemental File 16). These results indicate that the pony can exercise while wearing the z-bar shoe to manage uniaxial palmar pain.

## 3. Discussion

This report described the long-term application of a z-bar shoe during the exercise training regimen in a show jumping pony suffering from chronic uniaxial palmar pain. Foot pain was alleviated following z-bar shoeing based on uniaxial perineural anaesthesia. Moreover, it restored movement efficiency during the exercise training regimen while managing the palmar pain. Therefore, z-bar shoeing demonstrated potential for long-term wear for foot pain management during the exercise training regimen in a show jumping pony with chronic uniaxial palmar pain.

As reported previously, palmar pain could have arisen from various structural injuries at the palmar hoof region, including navicular bone and its associated apparatus, navicular bursa, the distal portion of deep digital flexor tendon, palmar part of the distal phalanx, and hoof capsule's palmar structure [2, 4]. Local anaesthesia has usually been introduced to narrow the pain location and facilitate clinical investigation in the affected limb [20, 21]. In this case, the pony showed pain in the medial palmar region in the right front hoof according to the uniaxial perineural anaesthesia. The pathological lesion radiographically detected in the navicular bone was unlikely to be associated with medial palmar pain. In addition, a similar lesion was seen in the navicular bone of the left front hoof, where no lameness was observed. A previous study reported that pathological changes in the navicular bone might be seen in the contralateral leg similar to the lame leg [4, 22]. Therefore, the source of pain resulting from the navicular bone could be excluded. The injuries of medial internal structures, including collateral ligament, hoof wall, and associated soft tissue, would account for the possible source of pain. According to the result of pain localisation, it desensitised all parts of the medial palmar region including partial desensitisation of the distal interphalangeal (DIP) joint and navicular bursa. The joint and bursa are less likely to be the cause of medial palmar pain due to the partial desensitisation following uniaxial regional anaesthesia. Moreover, doing the intrasynovial DIP joint anaesthesia for further diagnosis may not fully indicate the pain arising from the intra-articular structure of the joint. That is because the local anaesthesia agent administrated into the DIP joint could also desensitise the palmar digital nerve where they lie in close proximity to the palmar pouch of the DIP joint [23, 24]. In addition, diffusion of the anaesthetic agent from the DIP joint to navicular bursa may also be found leading to concomitant desensitisation of the bursa [25]. For this reason, the intrasynovial DIP joint and intrabursa anaesthesia were not performed in this case to avoid the possibility of false interpretation. Magnetic resonance imaging (MRI) has frequently been used for accurate diagnosis, including the injuries of deep digital flexor tendon or collateral ligament of the distal interphalangeal (DIP). One advantage of MRI is that it provides a prognosis of the treatment outcome [3, 26]. Unfortunately, a definite diagnosis could not be reached in this case as an MRI was not available. The management strategy was based on the evidence of uniaxial palmar pain regardless of knowing the exact structures from where the pain was derived.

Having reported that the principle treatment protocol, including rest, control exercise, and creative therapeutic shoeing, has been implemented to correct chronic heel pain [2]. In this case, only the z-bar shoe was employed as therapeutic shoeing for managing foot pain. The empty portion at the medial side was designed to unload the affected palmar region, thereby alleviating the existing palmar pain. As expected, the pony showed a marked improvement in lameness severity following the z-bar shoeing in the right front hoof throughout the shoeing period. In addition, no medical intervention was provided throughout the study. More importantly, the pony could undergo an exercise training program while wearing the Z-bar shoe, leading to the desired outcome of exercise performance during each period of shoeing adjustment (Table 1). These results suggest that long-term application of the z-bar shoe could manage foot pain and maintain exercise efficiency in a pony with uniaxial palmar pain. Concerning the balance of movement, a change in hoof balance may result in an uneven loading pattern [27], in which the steeper hoof is prone to generate more vertical force than the flatter hoof [28]. Accordingly, hoof conformation might be the predisposing cause of the trotting asymmetry during exercise training. In this pony, a slight asymmetry was detected during exercise training at 12 to 20 weeks of z-bar shoeing. Notably, the pony showed flatter hoof conformation in the left than the right front hooves (Figure 1). Interestingly, a marked improvement of trotting symmetry was observed following strategic trimming to restore hoof balance at 24 weeks of shoeing. Asymmetry may be expected following rising trot during exercise training [29, 30]. However, the pony was ridden with rising trot throughout the exercise training regimen. Thus, trotting symmetry improvement resulted mainly from the restoration of the hoof balance regardless of riding pattern. As restoration of hooves' balance, denoted by an improvement of the hoof-pastern axis on the left front hoof and reduced positive hoof angle right front hoof, was observed radiographically at 30 weeks of the shoeing management, it was plausible that the z-bar shoeing with the gap portion eliminated the weight-bearing on the hoof's medial palmar region, causing the weight shifting to a more lateral palmar region. This event would lead to an improvement of medial displacement of the proximal and intermediate phalanxes on the right front hoof as shown in Figure 5.

Despite the advantage of z-bar shoeing in the current case, using this method over a longer period in larger horses and during different training regimes requires validation with larger sample sizes.

## 4. Conclusion

Z-bar shoeing showed the potential of long-term application during an exercise training regimen while managing foot pain in a show jumping pony diagnosed with chronic uniaxial palmar pain. This report also provided the information that the therapeutic shoeing could be effectively performed based on the tentative diagnosis as uniaxial palmar pain even though the definite diagnosis could not be reached. A close collaboration between the farrier and the practitioners is still of paramount importance to achieve a favourable outcome.

## Figures and Tables

**Table 1:** The parameters obtained from the heart rate detection device during exercise training at 16, 20, and 24 weeks of z-bar shoeing.

Parameters	12 weeks	16 weeks	20 weeks	24 weeks
Maximal heart rate (bpm)	133	139	148	134
Average heart rate (bpm)	98	92	99	96
Intensity of training (%)	30	27	33	29
Maximal speed (km/h)	10.1	9.8	11.7	12.3
Average speed (km/h)	7.2	6.7	7.7	6.3
Total distance (km)	5.4	4.5	5.2	4.0
Duration (min)	44	42	44	39
Trotting symmetry (%)				
Left	47	46	47	51
Right	53	54	53	49
Average diagonal duration (s)				
Left	0.354	0.332	0.339	0.406
Right	0.394	0.397	0.381	0.385
Cadence (strides/min)				
Walk	49	48	48	53
Trot	85	85	86	88
Canter	109	111	108	111
Elevation (cm)				
Walk	3	3	3	3
Trot	4	5	5	4
Canter	14	14	14	14

Recommended value of cadence: walking, 35–60 strides/min; trotting, 55–100 strides/min; cantering, 80–130 strides/min. Recommended value of elevation: walking, 1–5 cm; trotting, 5–15 cm; cantering, 10–25 cm.

## Data Availability

Supplemental files 1–16 are available at https://drive.google.com/drive/folders/12Os7HWxHQbc1KmoOclck6KZlA7rs4yLm?usp=sharing.
