# Supplementary material for: Z-Bar Shoeing Demonstrates Potential for Long-Term Foot Pain Management during an Exercise Training Regimen in a Show Jumping Pony with Uniaxial Palmar Pain
Source: Case Rep Vet Med. 2022 Apr 13;2022:8468403. doi: 10.1155/2022/8468403 (PMC9020972; doi:10.1155/2022/8468403)
Supplement: Supplementary Materials — Supplemental files 1–16 are available at https://bit.ly/3va6UsB. [file 8468403.f1.docx]

**Supplementary materials**

Supplemental files 1-16 are available at <https://bit.ly/3va6UsB>
